# A comprehensive meta-analysis and prioritization study to identify vitiligo associated coding and non-coding SNV candidates using web-based bioinformatics tools

**DOI:** 10.1038/s41598-022-18766-9

**Published:** 2022-08-25

**Authors:** Tithi Dutta, Sayantan Mitra, Arpan Saha, Kausik Ganguly, Tushar Pyne, Mainak Sengupta

**Affiliations:** 1 Department of Genetics, University of Calcutta, 35 Ballygunge Circular Road, Kolkata, 700019 India; 2 Department of Genetics, CVM University, Aribas, Aribas Campus, New Vallabh Vidyanagar, Anand, Gujarat 388121 India

**Keywords:** Computational biology and bioinformatics, Genetics, Immunology, Pathogenesis, Risk factors

## Abstract

Vitiligo is a prevalent depigmentation disorder affecting around 1% of the general population. So far, various Genome Wide Association Studies (GWAS) and Candidate Gene Association Studies (CGAS) have identified several single nucleotide variants (SNVs) as a risk factor for vitiligo. Nonetheless, little has been discerned regarding their direct functional significance to the disease pathogenesis. In this study, we did extensive data mining and downstream analysis using several experimentally validated datasets like GTEx Portal and web tools like rSNPBase, RegulomeDB, HaploReg and STRING to prioritize 13 SNVs from a set of 291SNVs that have been previously reported to be associated with vitiligo. We also prioritized their underlying/target genes and tried annotating their functional contribution to vitiligo pathogenesis. Our analysis revealed genes like *FGFR10P*, *SUOX, CDK5RAP1 and RERE* that have never been implicated in vitiligo previously to have strong potentials to contribute to the disease pathogenesis. The study is the first of its kind to prioritize and functionally annotate vitiligo-associated GWAS and CGAS SNVs and their underlying/target genes, based on functional data available in the public domain database.

## Introduction

Vitiligo is the most prevalent acquired skin pigmentation disorder characterized by areas of progressive depigmented skin with a prevalence rate of 0.2% to 1% among various ethnic populations globally^1,^^2,^^3^. Several explanations have been proposed to explain the pathophysiology of vitiligo. The generally accepted mechanisms are lack of melanocytes due to destruction^4^, defects in melanocyte adhesion and innervation^5^, microvascular anomalies, oxidative stress, autoimmunity^6–8^, metabolic abnormalities in pigmentation pathway, generation of inflammatory mediators^9^,and genetic and environmental factors^2,7^. In a recent study, chemical or occupational-induced vitiligo was also found to occur^10^. There are two basic forms of vitiligo, based on the disease's classical traits and natural history. i.e. Segmental Vitiligo (SV) with a unilateral pattern of dermatomal distribution and Non-segmental Vitiligo (NSV) with an array of pigmentary disorders localized symmetrically. Recent studies have suggested that there is a shared mechanism between the two subtypes^11^.Vitiligo, again, is divided into three categories based on its distribution—localized, generalized, and universal^12^. Determining the proper subtype, thereby, might be essential for illness management and prognosis. Vitiligo prevalence was found to rise with age in a population-based study^12^, although no such association was found in other epidemiological investigations^1^.

It has been suggested that vitiligo susceptibility could be attributed to genetic variants associated with key regulatory pathways such as melanin biosynthesis^13^,*TLR* signaling^14^, apoptosis^15,^^16^,Vitamin D metabolism^17^, inflammatory and oxidative stress-responsive pathways^18,^^6,^^19^ etc. Several association studies, both at the genome-wide scale^20,^^21,^^22^ and at candidate gene level^14,23,24^ have identified many variants associated with vitiligo. The results of a vitiligo GWAS in the Han Chinese population identified rs10876864-*PMEL*, rs638893-*CXCR5/DDX6,* and rs11417210-*SLC29A3/CDH23* to be associated with vitiligo risk. rs10876864 and rs638893 have both been associated with autoimmune diseases implying that concurrent molecular pathways might contribute to vitiligo pathogenesis^21,^^25^. Genes involved in melanocytic stress such as *SOD2, GSTP1* and *XPB1* have also been identified by GWAS^6,^^7,^^26,^^27^. However, more often than not the functional annotation of the variant loci was done to reveal the molecular pathway behind vitiligo susceptibility. This is especially true for the associated SNVs in the non-coding regions, sometimes at a significant physical distance upstream or downstream from their nearest genes^28^. A candidate gene association study conducted on an English population comprising 165 patients and 304 controls found that rs2476601-T allele of *PTPN22* was significantly associated with generalized vitiligo ^29^ however it is unclear how the gene *PTPN22* contributes to vitiligo. According to another case–control study, conducted on 166 patients and 169 ethnically matched controls within the English population, rs769217 C/T heterozygotes of *CAT* gene showed a significant predisposition to vitiligo^30^, which, however, was not significant in the Korean population^31^. A functional annotation of the variant loci may help in understanding if the association was by chance or points towards a hitherto unexplained pathway to the disease, in these cases of contrary findings^32,^^33^. Therefore, we thought it is imperative to prioritize and assess the functional relevance of genomic variants that can play a role in vitiligo etiology.

While functional prediction of exonic variants through bioinformatic programs had been in use for quite some time, the advent of Encyclopedia of DNA Elements (ENCODE; https://www.encodeproject.org) and auxiliary datasets have now, made it possible to ascribe regulatory role for the non-coding SNVs associated with complex diseases like vitiligo.Genomic variants regulating DNA binding, transcription, DNA accessibility, RNA binding, DNA methylation, replication timing, genotyping, proteomics, DNA sequencing, RNA structures, etc., can be validated using these datasets generated experimentally from human bio-samples. In this context we thought identifying the actual vitiligo susceptibility conferring genetic variations and their corresponding pathways^34^ from a pool of the associated SNVs would provide further insights into the heritable predisposition of the disease in the world population^35^. This may provide novel therapeutic and even prophylactic targets for the prevention and treatment of vitiligo^33,^^36^.

We have designed and utilized a bioinformatics pipeline to prioritize variants associated with human pigmentation^34^ and subjective well-being^37^. We understand similar bioinformatic analysis has been done for other complex diseases like type II diabetes to prioritize SNVs from a pool of GWAS and CGAS based associated variants, and provide their functional relevance^38,^^39^. Here, through a similar study-design, we aim to prioritize and assess the potentially significant SNVs and their underlying/target genes in rendering vitiligo susceptibility. During the course of the study, we tried to determine how the prioritized variants and their loci contribute to vitiligo development. To our knowledge, this is the first study of its kind to prioritize and functionally annotate the vitiligo-associated SNVs using data primarily from the public domain database.

## Methodology

### Brief overview of analysis followed in our study

**Figure 1:**
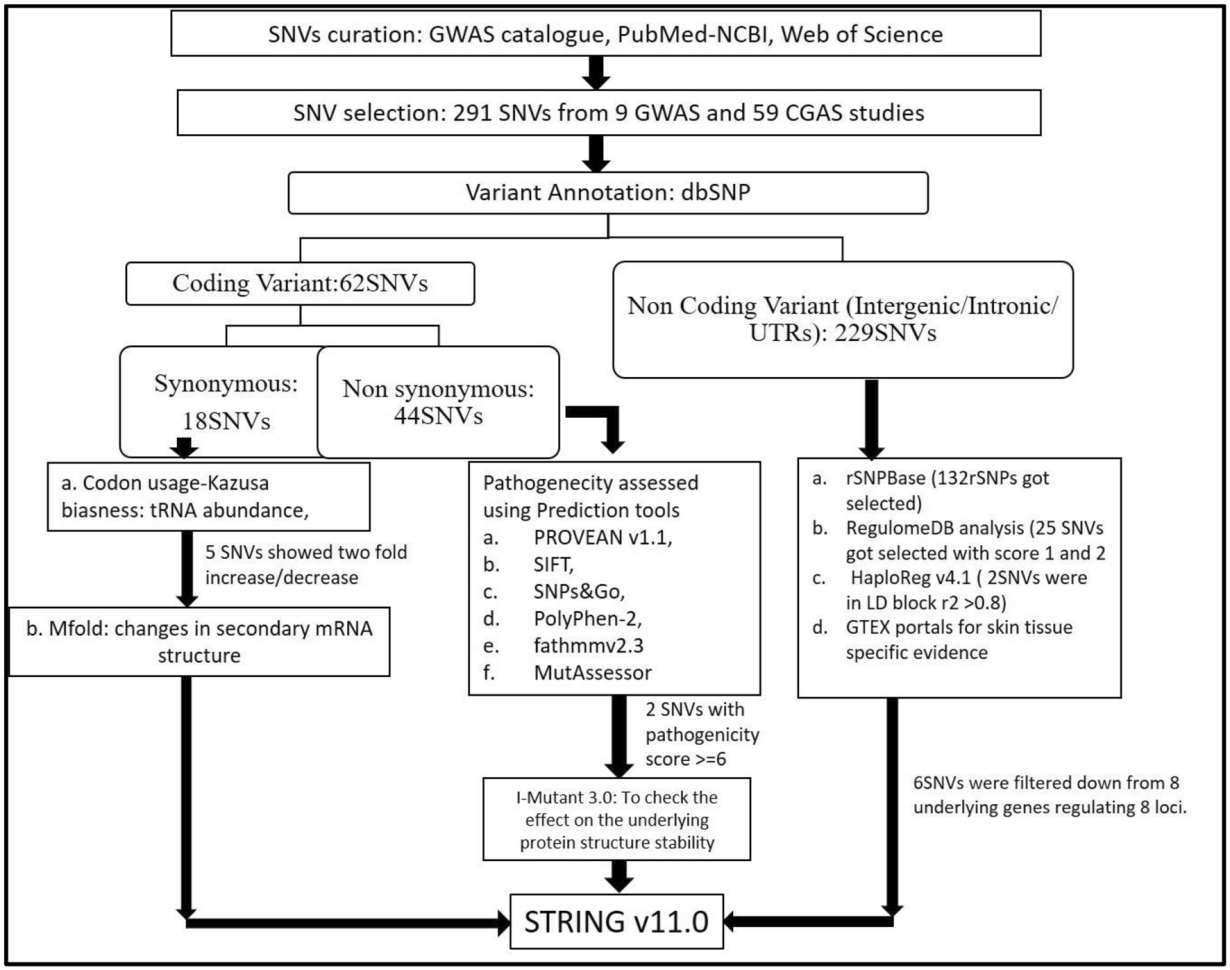
Scheme of the analyses used for prioritization of Vitiligo associated-SNVs. The SNVs and associated information were curated from GWA studies catalog, PubMed and Web of Science. They were categorized into coding and non-coding variants using dbSNP, and the coding variants were further classified into synonymous and non-synonymous. Using different bioinformatics programs the functional potential of each of these SNVs was identified and the SNVs were ranked on that basis. While the synonymous variants were prioritized based on their codon usage biasness and changes in predicted secondary mRNA structure, the non-synonymous variants were prioritized through programs assessing the ability of the variant amino acid to tinker the function of the protein concerned. The non-coding SNVs were assessed for their regulatory potential using several bioinformatics programs. The skin tissue specific regulatory role(s) were assessed through eQTL based evidence from GTEx Portal. Lastly, the genes in which the prioritized SNVs were located or the target genes of the prioritized SNVs were checked for their presence in Harmonizome, GeneMania and KEGG: Kyoto Encyclopedia of Genes and Genomes. Interaction between the proteins coded by the genes that were thus prioritized through the 3 priority pipelines viz. that for non-synonymous, synonymous and regulatory SNVs, was checked by STRING v11.0.

From the GWAS catalogue (https://www.ebi.ac.uk/gwas/), PubMed (https://pubmed.ncbi.nlm.nih.gov/), and Web of Science (https://login.webofknowledge.com/), a total of 68 GWAS and CGAS were included for analysis in our study. The SNVs were categorized into non-coding (regulatory) and coding (composed of non-synonymous and synonymous) SNVs using the dbSNP database (https://www.ncbi.nlm.nih.gov/snp/). Each of these SNVs was ranked according to their functional importance and probable roles in downstream gene regulation using three separate bioinformatics pathways used for non-coding, non-synonymous, and synonymous SNVs. PROVEAN v1.1, SIFT, SNPs&Go, PolyPhen-2, Fathmm v2.3, and MutAssessor were used to prioritize non-synonymous variations. The protein stability of those SNVs with deleterious consequences was subsequently evaluated using I-Mutant 3.0. The synonymous variations were prioritized based on their codon usage bias and their changes in secondary mRNA structure through the mfold Web Server.The regulatory potential of non-coding SNVs was examined using rSNPBase and RegulomeDB, followed by Linkage Disequilibrium (LD) analysis using Haploreg v4.1. By using eQTL based evidence from GTEx Portal, we tried to ascribe skin tissue specific regulatory role(s), if any, of the noncoding SNVs. STRING v11.0 was used to analyze protein–protein interactions based on the gene sets prioritized by all the priority pipelines of SNV selection. The overall analysis schema is depicted in Fig. 1.

### SNV curation and study design

To find the list of vitiligo associated SNVs, we searched GWAS catalogue (https://www.ebi.ac.uk/gwas/), PubMed (https://pubmed.ncbi.nlm.nih.gov/) and Web of Science (https://login.webofknowledge.com/) using the search string “Vitiligo AND/and Polymorphism/SNP/SNPs/SNV/SNVs AND/and India/World/Global”. After removing repetitions and common variants, we found 291 SNVs from 9 GWASs and 59 CGAS. Reports included in our study considered all types of vitiligo, namely, segmental, non-segmental, localized, generalized, universal, and mixed vitiligo. The finalized list of SNVs was grouped into A. Coding SNVs, B. Non-Coding SNVs using literature and the dbSNP database (https://www.ncbi.nlm.nih.gov/snp/). Coding SNVs were categorized further as A1. Non-Synonymous SNVs, A2. Synonymous SNVs. In order to prioritize the most significant SNVs and their underlying/target genes, we subjected each category of SNVs to different bioinformatics pipelines.

i. Analysis of non-synonymous SNVs:
  The non-synonymous SNVs were subjected to analyses through the following tools: PROVEAN v1.1, SIFT, SNPs&Go, PolyPhen-2, fathmm V2.3, MutAssessor and I-Mutant. The outcome of each tool for a specific SNV was assigned a unit score, where the higher the score, the more would be the non-neutral character of the SNV.
  PROVEAN or Protein Variation Effect Analyzer v1.1 (http://provean.jcvi.org/seq_submit.php), computes the prediction score for instance, if PROVEAN computes a SNV to be deleterious, we scored it as 1 and if the SNV is considered to have a neutral effect, we scored it as 0. SIFT classifies amino acid substitution as tolerant or deleterious viz if the SIFT Score is lesser than 0.05, the variant is predicted as damaging. The Coding SNVs were submitted in “Provean Batch Protein Tool” (http://provean.jcvi.org/index.php) under species “HUMAN” which provided us with both PROVEAN and SIFT prediction score. The consequence of the non-synonymous variant was then checked in SNPs&GO and PolyPhen-2.
  SNPs&GO (https://snps-and-go.biocomp.unibo.it/snps-and-go/) was used for predicting disease related mutation using GENE Ontology Annotation. HumVar PolyPhen-2 (http://genetics.bwh.harvard.edu/pph2/) was used to evaluate a variant qualitatively, as benign, possibly damaging or probably damaging and also provides an output of numerical score ranging from 0 being benign to 1 being damaging. Fathmmv2.3 (http://fathmmv2.3.biocompute.org.uk/) and Mutation Assessor (http://mutationassessor.org/r3/) were used to assess the functional implication of the non-synonymous variant based on evolutionary conservation of the affected amino acid in the protein homologue where positive fathmm v2.3 scores showed a tolerance to the variation (Output: Tolerated) whereas negative score showed intolerance to the pathogenic variation (Output: Damaging). Thus, we prioritized the SNVs and the underlying genes with a score of 6 from category A1 as shown in supplementary Table S4. Using I-Mutant 3.0, we tried to predict the possible implication on protein stability. I-Mutant 3.0 (http://gpcr2.biocomp.unibo.it/cgi/predictors/I-Mutant3.0/I-Mutant3.0.cgi) was used for determining the protein stability by calculating the free energy change (DDG or change in free energy G)upon inducing single site mutations via Binary prediction i.e. a positive DDG value indicates an increase in the stability of the protein whereas a negative DDG value indicates destabilisation of the protein. The FASTA sequence of the protein corresponding to the variant was submitted as input to predict the protein stability changes at a given pH and Temperature. Besides, it also provides Reliability Index (RI) value to assess the accuracy of prediction.Both the structure-based and sequence-based predictions of the change in protein stability upon mutation were assigned a reliability scoring index (RI), which allows the selection of the most reliable predictions. The RI ranges from 0 to 9, values from 0 to 5 indicate highly unstable proteins while 6–9 indicate high protein stability. The complete analysis of non-synonymous variants is tabulated as shown in Supplementary Table S4.
ii. Analysis of synonymous SNVs:
  The synonymous SNVs were analysed for their Codon Usage Biasness (following the Codon usage table available at https://www.kazusa.or.jp/codon/) to identify potential changes in codon redundancy inflicted due to presence of different allelic variants of same synonymous SNV. Due to the phenomenon of redundancy of the codon, specific anti-codon containing tRNAs are more available in living organisms than other codons and any synonymous nucleotide variation in codon sequence, thus, could alter protein synthesis but not the peptide sequence. In this study, a two-fold increase/decrease in tRNA abundance in between wild -type and mutant codon was taken into consideration as a significant change which were then analyzed in the mfold Web Server RNA folding form (http://unafold.rna.albany.edu/?q=mfold/RNA-Folding-Form). mfold Web Server provides us the predicted secondary mRNA structure based on query FASTA sequence containing wild-type and mutant SNV sequence along with ΔG value for the same representing protein stability. Following this, we checked each circular plot (as shown in Supplementary Fig. 1) and noted the change in the circular plot of the RNA secondary structure via the 'RNA Folding Form' of mfold Web Server. In the "View Individual Structures" section, the RNA Folding Form provides a hierarchical view of the possible stem-looped secondary structures based on delta G values, for all probable stem-looped secondary structures. The syn-SNVs whose circular plots were not different between the different allelic forms for a particular syn-SNV were filtered out. [see S6].
iii. Analysis of non-coding SNVs:
  For the non-coding SNVs, we wanted to assess their regulatory potential [i.e. ability to regulate transcription of the target gene] if any, and thus prioritize them along with their target genes with respect to their contribution towards developing vitiligo. We ran the SNVs through rSNPBase and RegulomeDB and selected the potential regulatory SNVs as predicted by these tools for further analysis.
  - rSNPBase
    The online regulatory database rSNPBase 3.0 provides functional evidence and expression quantitative trait loci (eQTLs) information for the query rSNV Ids, focusing on their regulation types like proximal, distal transcriptional regulation, RNA binding protein-mediated post-transcriptional regulation and identifies their potentially regulated genes. All the regulatory SNVs present under the non-coding category were submitted in enter delimited format by using the “List search” option of rSNPBase (http://rsnp.psych.ac.cn/). For each query ID, where the result page gave “YES” as the end result, provide a reliable experimentally proven regulatory annotation of the particular SNV Id. Whereas if the result page gave “NO” outcome, we excluded those SNV Ids for further analyses. In addition, rSNPBase has been used to investigate miRNA-mediated post transcriptional regulatory potential for SNVs mapped to the 3'UTR region.
  - Regulome DB 2.0
    Regulome DB helps us to identify putative regulatory potential of the variants having deleterious impact on gene regulation by including high-throughput, experimental data sets from ENCODE. The scoring system ranges from 1 to 6 based on integrating functional genomics features along with continuous values such as ChIP-seq signal, DNase-seq signal, information content change, and DeepSEA scores among all the variants. SNVs with positive outcome in rSNPBase were submitted in RegulomeDB 2.0 (https://regulomedb.org/regulome-search/) in the “dbSNP ID” option.
  - Haploreg v4.1
    Haploreg v4.1 is a freely available online web-tool that enables the user to identify query SNVs falling within the Linkage Disequilibrium (LD) blocks using chromatin state and protein binding capability from ENCODE project which shows the effect of the variants on regulatory motifs and probable role in expression based on eQTL data. Thus, it is important to mention, Haploreg analysis was not taken into consideration for prioritization of SNVs.

### Prioritization of regulatory SNVs using cell line based evidence

As a consequence of prioritizing the non-coding SNVs using the above-mentioned tools, we also identified their target genes through GTEx and checked if vitiligo's skin cells showed enough DNAse hypersensitivity signals. By using the hyperlinks in the "SNP Id" column in rSNPBase, we checked the type of regulation and the targets of the regulatory variants. Vitiligo is characterized by white patches and macules on the epidermal layer of the body. Therefore, to evaluate the regulatory signals of SNVs in skin-specific tissues (Keratinocyte, Fibroblast, Melanoma, Sun-exposed skin, non-sun-exposed skin), we analyzed them in the rSNPBase and GTEX Portal. The filtered SNVs obtained from ENCODE-based web-tools were then checked for skin-specific evidence using rSNPBase, which provides spatiotemporal labels on the tissue and developmental stage cell for regulatory elements. These SNVs were then analyzed and cross checked for skin specific eQTL based evidence in GTEx Portal (https://gtexportal.org/home/) to help us discern the gene expression and regulation pattern contributing to the SNVs in the targeted tissue. The SNVs that appear to regulate common regulatory loci/possible target genes in SKIN specific tissues in both rSNPBase and GTEx Portal have been selected for further analysis as can be seen in Supplementary Tables [S8-S11].Following that, all the prioritized genes derived from the three pipelines were checked in Harmonizome (https://maayanlab.cloud/Harmonizome/), KEGG: Kyoto Encyclopedia of Genes (https://www.genome.jp/kegg/pathway.html)^40^ and GeneMania (https://genemania.org/) to determine how many of them were already represented within the vitiligo pathway and if they had any protein–protein interactions [see S14].

#### Interactome analysis using STRING v.11.0

String database annotates the functional interaction between proteins contributing to a specific biological function through systematic co-expression analysis, computational prediction methods, automated text mining of scientific literature, and pathway-based information from manually curated databases (https://www.genome.jp/kegg/). Using the STRING database, we attempted to identify how many of the prioritized SNV harboring loci or related target genes are interacting with already known vitiligo causing genes. A biomolecular data integration web portal, Harmonizome Gene-Disease Associations dataset contains a list of 13 genes associated with the disease "Vitiligo". Using our query genes and the data from GeneMANIA database, a network of gene sets (already known) associated with human diseases can be generated. A dataset of function-characterized genes in vitiligo has also been collected from the KEGG GENES Database using the search term "vitiligo". In the Results section, the steps are described in detail.

## Results

Following the removal of common or repetitive SNVs, a final list of 291 SNVs was obtained from literature-based GWA and CGA studies. We finally tabulated all the SNV Ids curated from the literature search results at cut off of p*1 × 10^−5^ (See S1). Of the 291 SNVs, 62 were classified as coding SNVs and 229 as non-coding SNVs. A total of 62 coding SNVs have been identified, 44 of which are non-synonymous and 18 of which are synonymous. There were 229 non-coding SNVs, including 222 intergenic/intronic SNVs and 7 3'UTR SNVs. Based on the final list of SNVs, a pipeline was used to assess them (see Fig. 1). In the following paragraphs, we discuss the results of web-based predictive tools and how priority was assigned to select SNVs.

### Prioritization of non-synonymous variants

A total of 44 non-synonymous variants were curated from literature search and were assessed for their pathogenicity through PROVEAN v1.1, SIFT, SNPs&Go, PolyPhen-2, Fathmm v2.3, MutAssessor. Two variants: rs1801133/*MTHFR* and rs5742905/*CBS*, found to have a pathogenicity score of 6 [see Methods for the basis of scoring] were analysed using I-Mutant 3.0 to observe their effect on the underlying protein structure stability. We found that for rs1801133, T allele in *MTHFR* gene had a probable deleterious role that might be implicated with a decrease in protein stability whereas rs5742905-C allele in *CBS* gene had a probable deleterious role that might be implicated with an increase in protein stability. However, I-Mutant 3.0 analysis was not considered as a filtering criterion for prioritization of SNVs as represented in Table 1 [see S5].

**Table 1:** List of the prioritized vitiligo associated non-synonymous SNVs.

SNVs	Ancestral allele	Location	Gene	Amino acid	PROVEAN	SIFT	SNPS&GO	POLYPHEN	FATHMM	MUT ASSESOR	WEIGHTAGE SCORE	I-MUTANT
					Prediction (cutoff = − 2.5)	Prediction (Score)	Effect (RI)	Prediction	Prediction (Score)	Functional impact (FI Score) FI score		Stability (RI)
rs1801133	C	chr1:11,796,321	*MTHFR*	A263V	Deleterious (3.76)	Damaging (0.002)	Disease (6)	Probably damaging	Damaging (4.03)	Medium (2.9) 2.965	6	Decrease (0)
rs5742905	T	chr21:43,063,074	*CBS*	I278T	Deleterious (3.82)	Damaging (0.001)	Disease (8)	Probably damaging	Damaging (4.57)	Medium (2.8) 2.895	6	Increase (0)

After checking the 44 non synonymous variants using PROVEAN v1.1, SIFT, SNPs&Go, PolyPhen-2, fathmm v2.3, MutAssessor and I-Mutant, we finally found two potential non-syn-SNVs from two genes that are predicted to play potential roles in vitiligo.

### Prioritization of synonymous variants

Our data-mining enlisted 18 synonymous variants that were subjected to the Codon Usage Database (https://www.kazusa.or.jp/codon/). The analysis revealed two-fold increase in codon biasness for rs35677492-A as opposed to the wild type whereas 4 variants [rs8192291-T, rs17886350-C, rs3134942-C, rs291700-A] showed two-fold decrease in codon usage compared to their wild type codons [S7]. These 5 variants were next checked in mfold that revealed major structural change for rs35677492, rs8192291, rs17886350, rs3134942, rs291700 as represented in Table 2 [see Supplementary Fig. 1].

**Table 2:** List of the prioritized vitiligo associated synonymous SNVs.

SNV	Location	Gene	cDNA position	Amino acid position	Codon	Codon usage biasness		W/M	ΔG value	
						% abundance of tRNA for wild-type(W) allele-based codon	% abundance of tRNA for mutant (M) allele-based codon		Wild type	Mutant
rs8192291	chr4:24,799,732	*SOD3*	306	71	CTG/TTG	39.6(1,611,801)	12.9(525,688)	0.32	− 138.9	− 138.9
rs17886350	11:34,456,025	*CAT*	806	242	ATC/ATA	20.8(846,466)	7.5(304,565)	0.36	− 66.41	− 61.19
rs35677492	chr11:34,471,409	*CAT*	1640	520	GCG/GCA	7.4(299,495)	15.8(643,471)	2.13	66.9	66.9
rs3134942	chr6:32,200,994–32,200,994	*NOTCH4*	1287	429	GTC/GTA	14.5(588,138)	7.1(287,712)	0.48	− 126.49	− 130.49
rs291700	chr20:33,394,043–33,394,043	*CDK5RAP1*	586	144	ACA/ACG	15.1(614,523)	6.1(246,105)	0.4	− 79.61	− 81.08

After checking the 18 synonymous variants using codon usage biasness followed by the changes in their secondary mRNA structure using mfold Web Server, we finally found four potential syn-SNVs from five genes that are predicted to play potential roles in vitiligo.

### Prioritization of non-coding variants

The 229 noncoding SNVs were analysed in rSNPBase to identify the 132 rSNPs that are 'Regulatory Single Nucleotide Polymorphisms'. In order to evaluate their regulatory potential, 132rSNVs were submitted to Regulome DB. We identified 13 rSNVs with score 1 [1b, 1c, 1d, 1f.] and 12 rSNVs with score 2 [2a, 2b, 2c]. A total of 24 rSNVs were characterized as intergenic/intronic rSNVs and one as 3'UTRs. The prioritized SNVs, identified to have probable regulatory role based on rSNPBase and RegulomeDB analysis were submitted in HaploReg in enter delimited format and LD threshold value was set to 0.8 (r^2^ > 0.8) while all other set options were kept as default. Our aim here was to only identify potential LD blocks among our query SNVs. In total, 25 rSNVs were found, of which two were in the LD block i.e.rs4750012 and rs925597 were found to be in LD with rs7099083 and rs925598, respectively with r^2^ value of greater than 0.8, thus we included rs4750012 and rs925597 only for our downstream analysis, which got filtered out as it did not reveal skin tissue based regulation in rSNPBase and GTEx portal respectively. rSNPBase demonstrated that 15 of these 23rSNVs target 92 loci using different modes of regulation [like proximal transcriptional regulation, distal transcriptional regulation, and RNA binding protein-mediated regulation], while no loci were found for the remaining 8 SNVs. Next, we tested these 15 SNVs for tissue-specific eQTLs using the GTEx portal. Finally, out of 229 noncoding SNVs, six SNVs were filtered down showing common regulatory target gene[s]/loci and skin-specific tissue expression based on rSNPBase and GTEx Portal from 8 underlying genes regulating 8 loci namely *CAT, RNASET2, PMEL-IKZF4 SUOX, RERE, FGFR10P and CCR6* as represented in Table 3 [See S14].

**Table 3:** List of the prioritized vitiligo associated non-coding SNVs.

Variant	Gene	Consequence	Alleles	*P* value from GTEx(tissue specific)
rs7943316	*CAT*	5' Upstream Transcript Variant	A>T	3.60E−20
rs2236313	*RNASET2*	Upstream	T>C	2.00E−20
rs10876864	*PMEL-IKZF4-SUOX*	Intron	G>A	4.00E−54
rs301807	*RERE*	Intron	A >G	2.50E−20
rs2247314	*RNASET2-FGFR1OP-CCR6*	5'near gene	T >C	1.10E−49
rs1701704	*IKZF4*	Intron variant	T > G	0.00021

After checking the 229 non-coding variants using rSNPBase and RegulomeDB, followed by LD analysis using Haploreg v4.1 and eQTL based tissue specific GTEx evidences, we finally found six potential regulatory SNVs from eight genes that are predicted to play potential roles in vitiligo.Two of our regulatory SNVs, i.e. rs108776864 and rs2247314 regulate multiple targeted genes as predicted by literature and rSNPBase.

It was intriguing that non-coding SNVs without any regulatory role as revealed by the low scores (6, 7) from RegulomeDB would be significantly associated with vitiligo. We thought a plausible explanation might be a SNV in LD with this non-regulatory SNV might be the affector variant in this regard that might have been missed in the concerned association study. So that we don’t miss any such SNVs we looked for the LD SNVs for such non-coding SNVs with very low RegulomeDB scores. We then selected 20 variants having regulomeDB scores 6 and 7 and submitted them to HaploReg v4.1, which found 380 LD SNVs, [as mentioned in Methodology], of which 12 LD SNVs with score 2 were considered potential regulatory roles. After following the above-mentioned pipeline, these 12 SNVs were checked in the rSNPBase and GTEx portals for tissue specific evidence. Based on our prioritization study, all twelve SNVs were filtered out as none had skin tissue specific evidence in both rSNPBase and GTEx portal. [see Supplementary A].

### Interactome analysis

**Figure 2:**
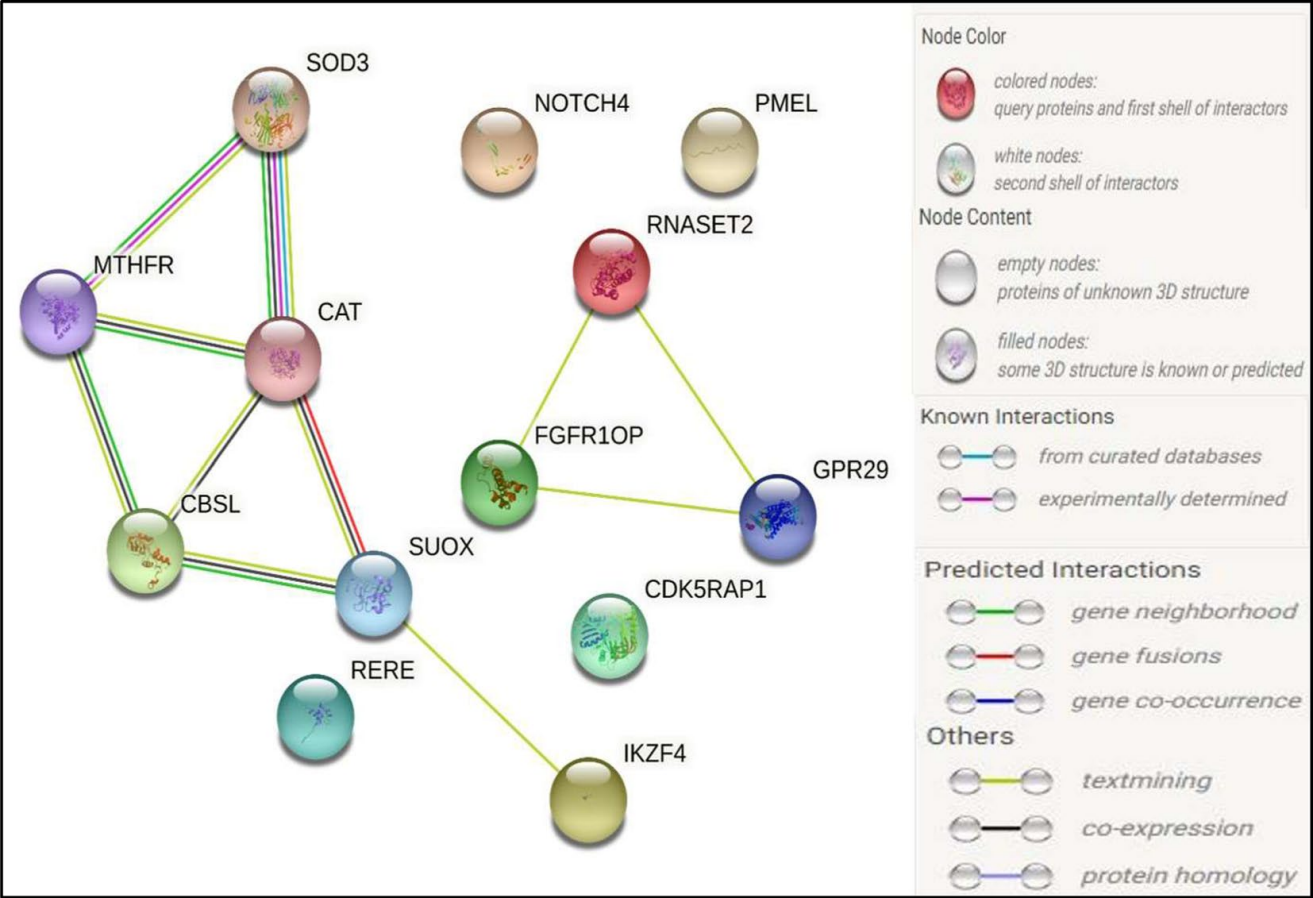
The figure illustrates the protein–protein interaction between the prioritized genes obtained from STRING v.11.0.

A final list of 13 SNVs was generated from a total of 291 SNVs initially selected from all searches, of which 62 SNVs were classified as Coding and 229 as Non-coding variants. These 13 prioritized SNVs were annotated to 13 target genes after eliminating duplicates. From the 13 selected targeted genes, *CAT* was found to be a common gene prioritized by both syn-SNV and non-coding SNV pipeline analyses. We wanted to assess if the 13 prioritized genes could interact with each other. We also wanted to check if these were represented in and/or had functional relationship with 194 well-known proteins associated with Vitiligo, as curated from the GeneMania, Harmonizome, and KEGG GENES databases. Thus, we ran STRING first with the 13 prioritized genes exclusively (Fig. 2) and then along with 194 genes (Supplementary Fig. 2) as mentioned above. STRING represents protein interaction by using light and dense network edges where light network edges depict low interaction confidence, and dense network edges depict high interaction confidence. Our prioritization pipeline revealed genes like *IKZF4* and *CCR6* that are already well-characterized to play a significant functional role in the development of vitiligo as evident from Harmonizome Dataset and GeneMania, thus indicating reliability and reproducibility of our prioritization process. The network suggested that 9 out of 13 prioritized genes: *CAT, RNASET2, CCR6, MTHFR, CBS, SOD3, SUOX, IKZF4 and FGFR1OP* showed interaction within themselves [Medium range of confidence score > 0.4] as represented in Fig. 2. Again, out of the 13 prioritized genes, 11 genes (*CAT, RNASET2, CCR6, MTHFR, CBS, SOD3, SUOX, IKZF4, FGFR1OP, NOTCH4 and PMEL)*showed interaction with at least one of the 194 genes implicated in vitiligo and documented in GeneMania, KEGG GENE Database and Harmonizome (Supplementary Fig. 2). Interestingly, two of our prioritized genes viz. *NOTCH4* and *PMEL* that were not represented in any of the 3-above mentioned datasets associated with vitiligo, were found to interact with well-known candidate genes like *FOXP1, STAT1, GPR29, TNF, CTSB* and many more. (Supplementary Fig. 2). STRING v11.0 analysis did not find *CDK5RAP1* or *RERE* in the protein interactome despite being prioritized following our syn-SNV and non-coding SNV study pipelines.

## Discussion

Globally, vitiligo is the most common complex acquired pigmentary disorder, and it is assumed to be partially penetrant due to gene–gene and gene-environment interactions. Genome-wide analysis and functional candidate gene association studies in vitiligo have discovered genetic variables that influence susceptibility^36^. Several GWAS over the past decade have led to the identification of SNVs that are statistically associated with vitiligo; nevertheless, the functional consequences of those associated polymorphisms have largely remained unknown^41^. In addition, GWAS indicates associated SNVs are dependent upon a strict statistical cut-off, and variants that do not make the cut-off are not considered, even if those genes have true biological meaning. This is especially relevant when considering the multifactorial aetiology and its combined effects on vitiligo pathogenesis^33^. We prioritised and identified the relevant SNVs and underlying/target genes contributing to vitiligo susceptibility using a comprehensive *in-silico* technique. From 291 SNVs collected from GWAS and CGAS, we identified 13 SNVs related with 13 genes to be potentially critically important for vitiligo pathogenesis. While 9 out of the 13 prioritized genes showed to interact with each other, 11 revealed interactions with at least one of the genes previously documented to have implications in vitiligo. This again, indicates the reliability of the prioritization process that points towards a complex network of genes that are associated with vitiligo pathogenesis.

We made an effort to functionally annotate the thirteen prioritized variants along with the underlying genes in vitiligo as represented in Table 4. Three of our prioritised genes: *CAT*^42^, *RNASET2*^43–45^, and *SOD3*^46^ are essential for physiological responses to oxidative stress caused by ROS which can lead to immune response and can result in melanocyte death, if deregulated. *CCR6* that codes for a receptor, when binds to chemokine ligands might promote melanocyte specific cytotoxic T lymphocytes to attack the melanocyte cells resulting in the deficiency of melanin and thus contributing to disease development^47,48^. *IKZF4* regulates T-cell transcription and inhibits the T-regulatory cell which are crucial to the development of self-tolerance and breakdown in immunological self-tolerance leads to aberrant immune response against melanocytes, contributing to the disease pathogenesis^21^. *MTHFR* and *CBS* are the major determinants of homocysteine metabolism and studies have shown significantly higher level of homocysteine in vitiligo patients^49,50^. *NOTCH4* affects the differentiation, proliferation, and regeneration of melanocytes during hair follicle cycles and retinal pigment epithelium development^51^ and *PMEL* plays a central role in the biogenesis of melanosomes involving the maturation of melanosomes from stage I to II which is an important event in the pathway of melanogenesis, implicating the role in vitiligo^21^. *FGFR1OP* and *SUOX*, have never been implicated towards vitiligo risk or pigmentation previously. *FGFR10P*, or Fibroblast Growth Factor Receptor 1 Oncogene Partner, regulates cell cycle progression^36,47^, and alterations of the gene have been important with regards to T-cell immunity. Studies have shown that vitiligo T cells display superior reactivity towards self-antigens resulting into progressive loss of melanocytes. *SUOX* is involved in sulfur metabolism i.e. conversion of sulphite to sulphate which if accumulated can result in oxidative stress and melanocytic cell death^21,46^. Interestingly, these two genes have shown interaction with known vitiligo associated genes like *YWHAE, HSP90AA, CTSB* and many more. On the contrary, *CDK5RAP1* and *RERE* have neither been previously implicated in vitiligo and/or pigmentation or were found to interact with known vitiligo related genes or any of the other prioritized genes. Cyclin-dependent kinase 5 regulatory subunit associated protein 1 (*CDK5RAP1*), is critically involved in the migration of neuroblasts during early post-natal development. The dysfunction of the sympathetic nervous system might affect melanogenesis causing depigmentation, supporting its role following the neuronal theory of the pathogenesis of vitiligo^52^. *RERE* is a transcriptional co-repressor which is highly expressed in lymphoid cells and is thought to trigger apoptosis when overexpressed^41^. Lymphoid cells, mainly dendritic cells, recognize pigmented cells as foreign and kill T cells, which results in vitiligo. So, RERE could also be considered as a risk factor for vitiligo^53,54^.

**Table 4:** Functional annotation of the thirteen prioritized variants along with the underlying genes in vitiligo.

SNVs	Gene within which the SNVs reside or genes they target	Function in human	Potential connection with vitiligo	Reported association with vitiligo from GWAS and CGAS
rs1801133	*MTHFR*	It catalyzes the conversion of 5,10-methylenetetrahydrofolate to 5-methyltetrahydrofolate, a co-substrate for homocysteine remethylation to methionine	*MTHFR* plays an important role in Homocysteine (Hcy metabolism). Elevated homocysteine level may be a precipitating factor for vitiligo	^49^
rs5742905	*CBS*	The enzyme hydrolase catalyses the first step of the transsulfuration pathway, where the hydroxyl group of L-serine is displaced by L-homocysteine in a beta-replacement reaction to form L-cystathionine, the precursor of L-cysteine. This catabolic route allows the elimination of L-methionine and the toxic metabolite L-homocysteine	Cystathionine B synthase is a major determinant of the homocysteine metabolism. Increased level of local Hcy interferes with normal melanogenesis pathway, thus lead to the disease precipitation	^49,50^
rs8192291	*SOD3*	Protect the extracellular space from toxic effect of reactive oxygen intermediates by converting superoxide radicals into hydrogen peroxide and oxygen	*SOD3* is essential for physiological responses to oxidative stress caused by ROS which can lead to immune response. Excessive accumulation of ROS can result in melanocyte death	^46^
rs17886350, rs35677492, rs7943316	*CAT*	Catalase is present in all aerobically respiring organisms and serves to protect cells from the toxic effects of hydrogen peroxide. Promotes growth of cells including T-cells, B-cells, myeloid leukemia cells, melanoma cells, normal and transformed fibroblast cells	Absence of catalase leads to the accumulation of ROS and creates oxidative stress, that has been suggested to be the initial pathogenic event in melanocyte degeneration with H2O2 accumulation in the epidermis of patients in	^42,53^
rs3134942	*NOTCH4*	It acts as a receptor for membrane-bound ligands Jagged1, Jagged2 and Delta1 to regulate cell-fate determination. Upon ligand activation through the released notch intracellular domain (NICD) it forms a transcriptional activator complex with RBPJ (Recombination Signal Binding Protein for immunoglobulin Kappa J region) and activates genes of the enhancer of split locus. It affects the implementation of differentiation, proliferation and apoptotic programs. It may regulate branching morphogenesis in the developing vascular system	*NOTCH4* affects the differentiation, proliferation, and regeneration of melanocytes during hair follicle formation and retinal pigment epithelium development	^54^
rs291700	*CDK5RAP1*	Methylthiotransferase that catalyzes the conversion of N6-(dimethylallyl) adenosine (i6A) to 2-methylthio-N6-(dimethylallyl) adenosine (ms2i6A) at position 37 (adjacent to the 3'-end of the anticodon) of four mitochondrial DNA-encoded tRNAs (Ser, Phe, Tyr and Trp). It specifically inhibits CDK5 (Cyclin Dependent Kinase) activation by CDK5R1	*CDK5RAP1* is critically involved in the migration of neuroblasts during early post-natal development, supporting its plausible role in vitiligo following the neuronal theory of the disease pathogenesis	^52^
rs2236313, rs2247314	*RNASET2*	Ribonuclease plays an essential role in innate immune response by recognizing and degrading RNAs from microbial pathogens that are subsequently sensed by TLR8. Cleaves preferentially single-stranded RNA molecules between purine and uridine residues, which critically contributes to the supply of catabolic uridine and the generation of purine-2′,3′-cyclophosphate-terminated oligoribonucleotides. In turn, RNase T2 degradation products promote the RNA-dependent activation of TLR8	*RNASET2* might act as an ‘‘alarm signal’’ in the development of vitiligo. It is essential for the physiological responses to oxidative stress caused by ROS which can lead to immune response and can result in melanocyte death, if deregulated	^43–45^
rs10876864	*PMEL*	Plays a central role in the biogenesis of melanosomes. Involved in the maturation of melanosomes from stage I to II. The transition from stage I melanosomes to stage II melanosomes involves an elongation of the vesicle, and the appearance within of distinct fibrillar structures. Release of the soluble form, ME20-S, could protect tumor cells from antibody mediated immunity	*PMEL* plays a central role in the biogenesis of melanosomes and maturation of melanosomes, which is an important event in the pathway of melanogenesis, implicating the role in vitiligo	^21,25^
	*SUOX*	This protein is involved in the pathway of sulfur metabolism, which catalyses the oxidation of sulphite to sulphate. It is localised in the intermembrane space of mitochondria, thus, change in SUOX might indicate oxidative stress and mitochondrial dysfunction	*SUOX* is involved in sulfur metabolism, which if accumulated can result in oxidative stress and melanocytic cell death	^21,53^
rs301807	*RERE*	It acts as a transcriptional repressor during development. May play a role in the control of cell survival. Overexpression of RERE recruits BAX (Bcl2-associated X Protein) to the nucleus particularly and triggers caspase-3 activation, leading to cell death	*RERE* which is highly expressed in lymphoid cells can trigger apoptosis when overexpressed, supporting its role in vitiligo following the autoimmune hypothesis of the disease pathogenesis	^41,54^
rs2247314	*FGFR1OP*	Fibroblast growth factor receptor 1 oncogenic partner or *FGFR1OP* may be involved in wound healing pathway. The protein can binds both acidic and basic fibroblast growth factors and promotes mitogenesis and differentiation	As alterations of the *FGFR1OP* have been found to affect T-cell immunity and studies have shown that vitiligo T cells display superior reactivity towards self-antigens resulting into progressive loss of melanocytes, *FGFR1OP* might contribute to disease pathogenesis	^47,48^
	*CCR6*	*CCR6* binds to CCL20 and subsequently transduces a signal by increasing the intracellular calcium ion levels. Although CCL20 is its major ligand it can also act as a receptor for non-chemokine ligands such as beta-defensins. The ligand-receptor pair CCL20-CCR6 is responsible for the chemotaxis of dendritic cells (DC), effector/ memory T-cells and B-cells and plays an important role at skin and mucosal surfaces under homeostatic and inflammatory conditions, as well as in pathology, including cancer and various autoimmune diseases	*CCR6* when binds to chemokine ligands might promote melanocyte specific cytotoxic T lymphocytes to attack the melanocyte cells resulting in the deficiency of melanin and thus contributing to disease development	^47,48^
rs1701704, rs10876864	*IKZF4*	It interacts with SPI1 (Spi-1 Protooncogene) and MITF (Melanocyte inducing Transcription Factor) to repress transcription of the CTSK (Cathepsin K gene) and ACP5 (Phosphatase 5 tartrate resistant acid) promoters via recruitment of corepressors SIN3A (paired amphipathic helix protein Sin3a) and CTBP2 (C-terminal binding protein 2). Essential for the inhibitory function of regulatory T-cells (Treg). Mediates FOXP3-mediated (Forkhead box P3) gene silencing in regulatory T-cells (Treg) via recruitment of corepressor CTBP1	*IKZF4* regulates T-cell transcription and inhibits the T-regulatory cell which is crucial to the development of self-tolerance and breakdown in immunological self-tolerance leads to aberrant immune response against melanocytes, contributing to the disease pathogenesis	^21^

## Conclusion

Being a visible skin disfiguring disease, vitiligo has a profound impact on the psychological and social aspects of the affected person. As a result, it was ranked as one of the major medical problems in India, along with leprosy and malaria. GWA studies and candidate gene association studies have successfully identified genetic variants associated with vitiligo risk, rarely defining their functional roles in driving vitiligo. Due to absence of reliable functional information about these variants and their harbouring genetic loci, researchers fail to extend their data for molecular diagnosis, further. In order to better define the complex nature of vitiligo, we tried to provide functional roles of the current GWA and CGA-identified nucleotide variants based on various omics data (generated using functional experimentations on patient derived tissue samples) available on public domain. In our study, we developed a bioinformatics pipeline using freely available web tools and tried to assign functional attributes to all the prioritized SNVs associated with vitiligo. Our study revealed *CBS, SOD3, CAT, RNASET2, SUOX, IKZF4, MTHFR, FGFR10P, PMEL*, *SUOX, NOTCH4, CDK5RAP1* and *CCR6* as candidates contributing towards vitiligo pathogenesis. Based on our prioritization pathway, we found that genetic variants of *FGFR10P, SUOX, CDK5RAP1 and RERE* have the potential to directly or indirectly contribute to the disease pathogenesis, suggesting that hitherto unexplored biological pathways may be involved in imparting vitiligo risk. To our knowledge, this is the first study of its kind that prioritized and functionally annotated the SNVs associated with vitiligo, largely based on functional data available in the public domain. The knowledge of the potential functional SNVs identified, along with their underlying genes, would pave the way for future studies to reveal previously unknown biological processes contributing to complex etiological outcomes of vitiligo. We believe that the prioritized nucleotide variants would enrich the current knowledge available for molecular diagnosis of vitiligo. Furthermore, the *in-silico* strategy employed for prioritization of genetic variants and their loci may be extended to other complex phenotypes, too. However, to ascertain the real-life involvement of prioritized genetic variants and their associated loci in development of vitiligo pathogenesis, further cell-line based or animal model based experimental validations are necessary.

## Supplementary Information

Supplementary Information 1.

Supplementary Information 2.

Supplementary Information 3.

Supplementary Information 4.

## Data Availability

All data generated or analysed during this study are included in this article. The bioinformatics tools used for the study are freely available web-based tools.

## Abbreviations

GWAS: Genome Wide Association Studies
CGAS: Candidate gene Association Studies
SNV: Single nucleotide variant
rSNPBase: Regulatory single nucleotide polymorphism base
GTEx: Genotype tissue expression
STRING: Search tool for the retrieval of interacting genes/proteins
ENCODE: Encyclopedia of DNA elements
TLR: Toll like receptor
MAPK: Mitogen-activated protein kinases
SOD: Superoxide dismutase
CXCR5/DDX6: Chemokine receptor type 5/DEAD-box helicase 6
SLC29A3/CDH23: Solute Carrier Family 29 Member 3/cadherin-like gene23
CAT: Catalase
GSTP1: Glutathione S-transferase pi gene
XPB1: X-box binding protein 1
PTPN22: Protein tyrosine phosphatase non-receptor type 22
RNASET2: Ribonuclease T2
FGFR1OP: Fibroblast growth factor receptor 1 oncogene partner
TLR6: Toll like receptor 6
MTHFR: Methylenetetrahydrofolate reductase
CBS: Cystathionine beta-synthase
PMEL: Premelanosome protein
IKZF4: IKAROS family zinc finger 4
SUOX: Sulfite oxidase
RERE: Arginine-glutamic acid dipeptide repeats
CCR6/GPR29: C-C motif chemokine receptor 6
UTR: Untranslated region
NOTCH4: Neurogenic locus notch homolog 4
KEGG: Kyoto encyclopedia of genes and genomes
FOXP1: ForkHead Box P1
STAT1: Signal transducer and activator of transcription 1
TNF: Tumor necrosis factor
CTSB: Cathepsin B
YWHAE: Tyrosine 3-monooxygenase
HSP90AA: Heat shock protein 90 alpha family class A
