# Supplementary material for: A comprehensive meta-analysis and prioritization study to identify vitiligo associated coding and non-coding SNV candidates using web-based bioinformatics tools
Source: Sci Rep. 2022 Aug 25;12:14543. doi: 10.1038/s41598-022-18766-9 (PMC9411560; doi:10.1038/s41598-022-18766-9)
Supplement: Supplementary file 1 — Supplementary Information 1. [file 41598_2022_18766_MOESM1_ESM.pdf]

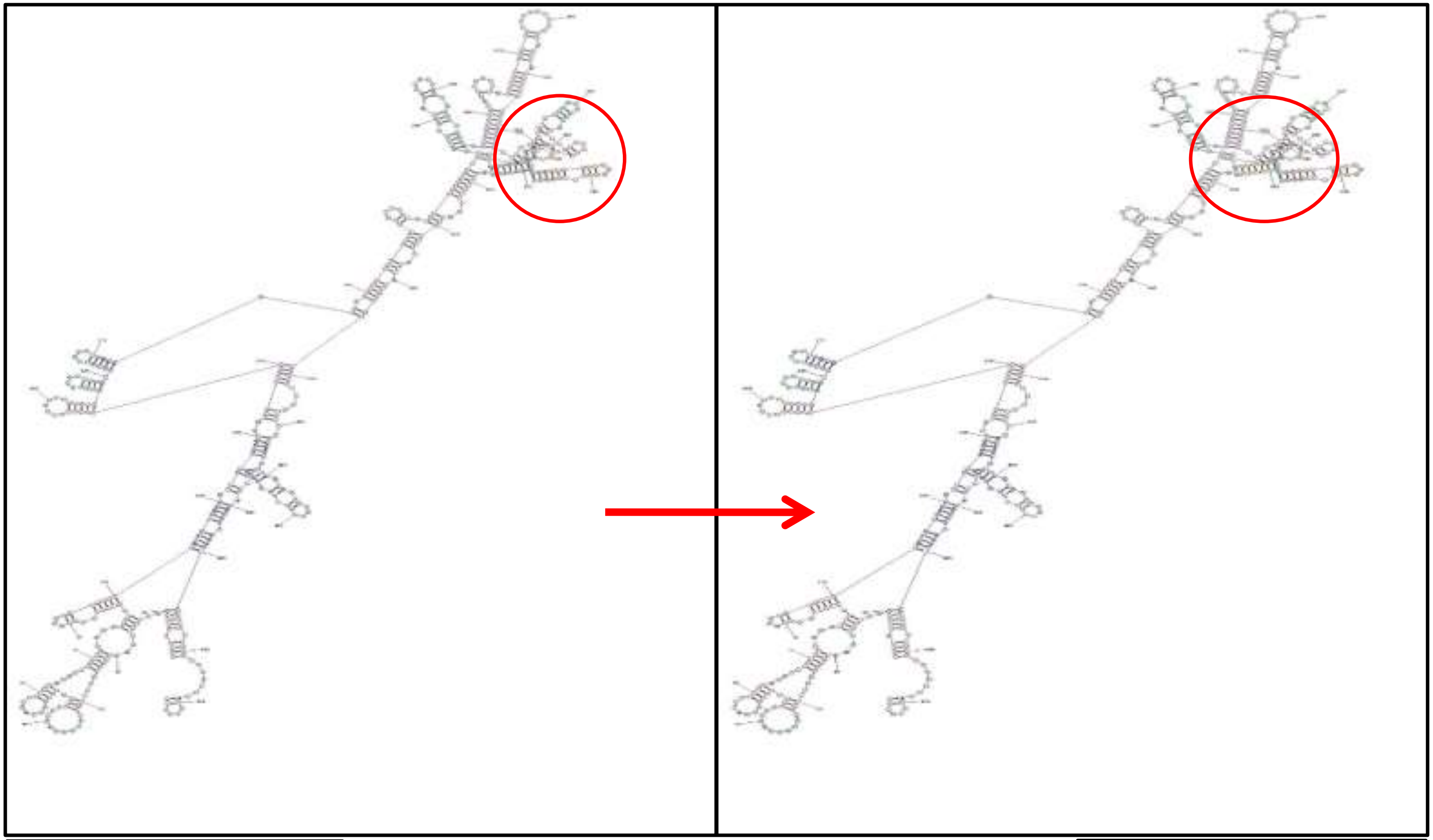

Energy= -127.8 [WT]

Energy= -127.8 [MT]

rs8192291 C>T_*SOD3*

Supplementary Figure 1 [SF1]: Pictorial representation of change in secondary structure of mRNA of *SOD3*gene due to rs8192291- wild-type [WT] and mutant [MT] variants.

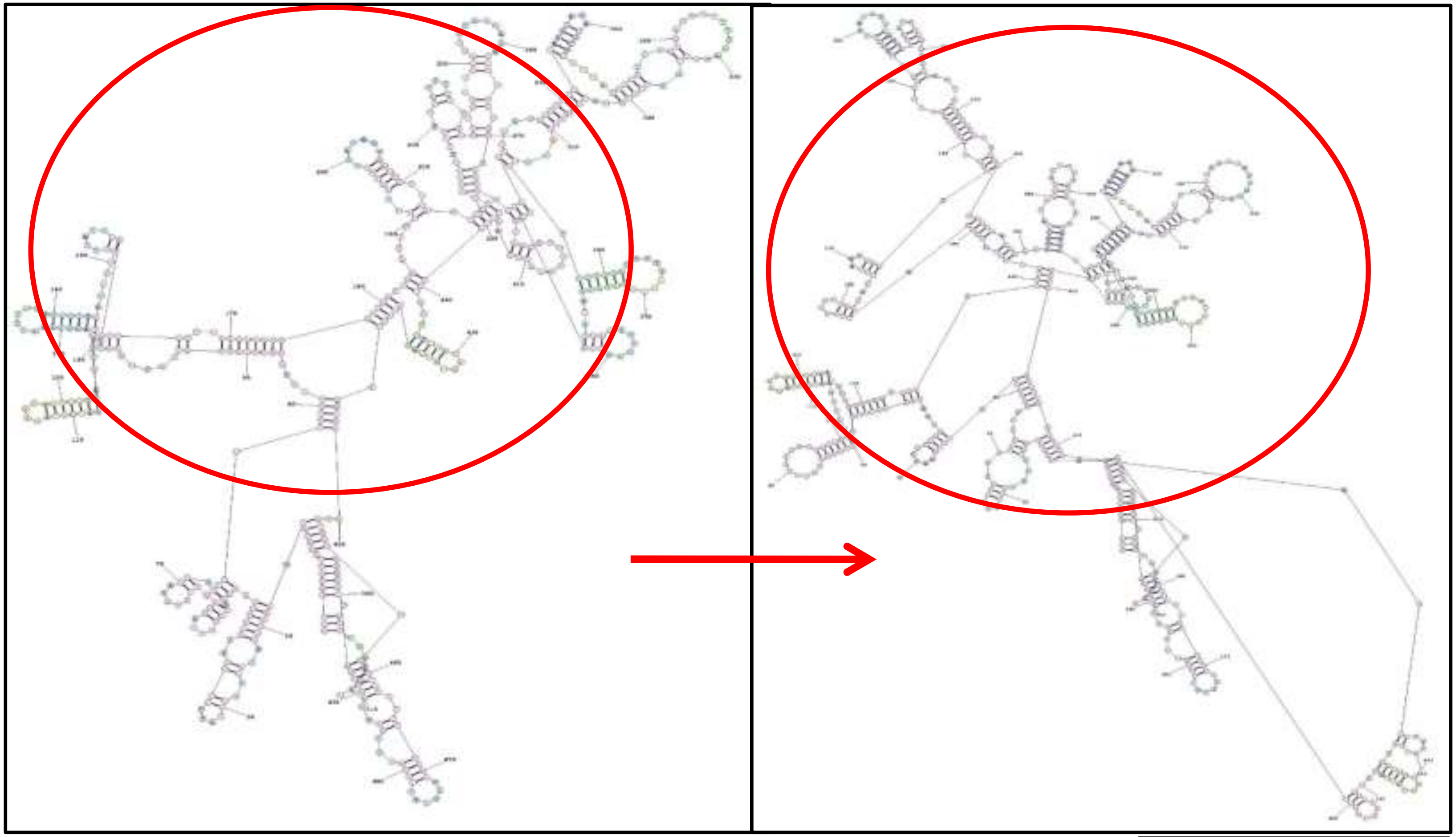

Energy= -55.6 [WT]

Energy= -57.6 [MT]

rs17886350A>C _*CAT*

Supplementary Figure 2 [SF2a]: Pictorial representation of change in secondary structure of mRNA of *CAT* gene due to rs17886350 wild-type [WT] and mutant [MT] variants.

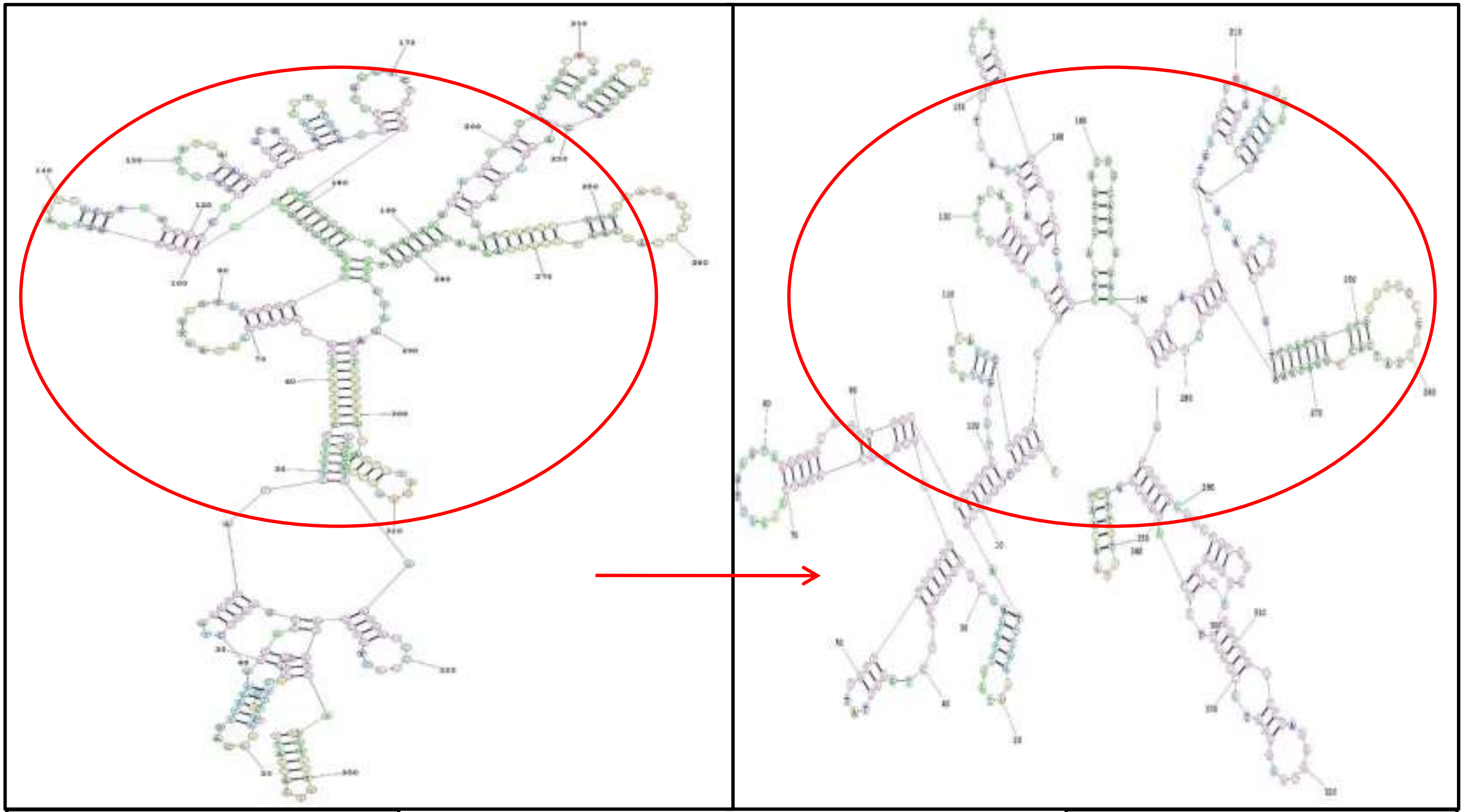

Energy= -54.0 [WT]

Energy= -53.4 [MT]

rs35677492 A>G_*CAT*

Supplementary Figure 2 [SF2b]: Pictorial representation of change in secondary structure of mRNA of *CAT* gene due to rs35677492 wild-type [WT] and mutant [MT] variants.

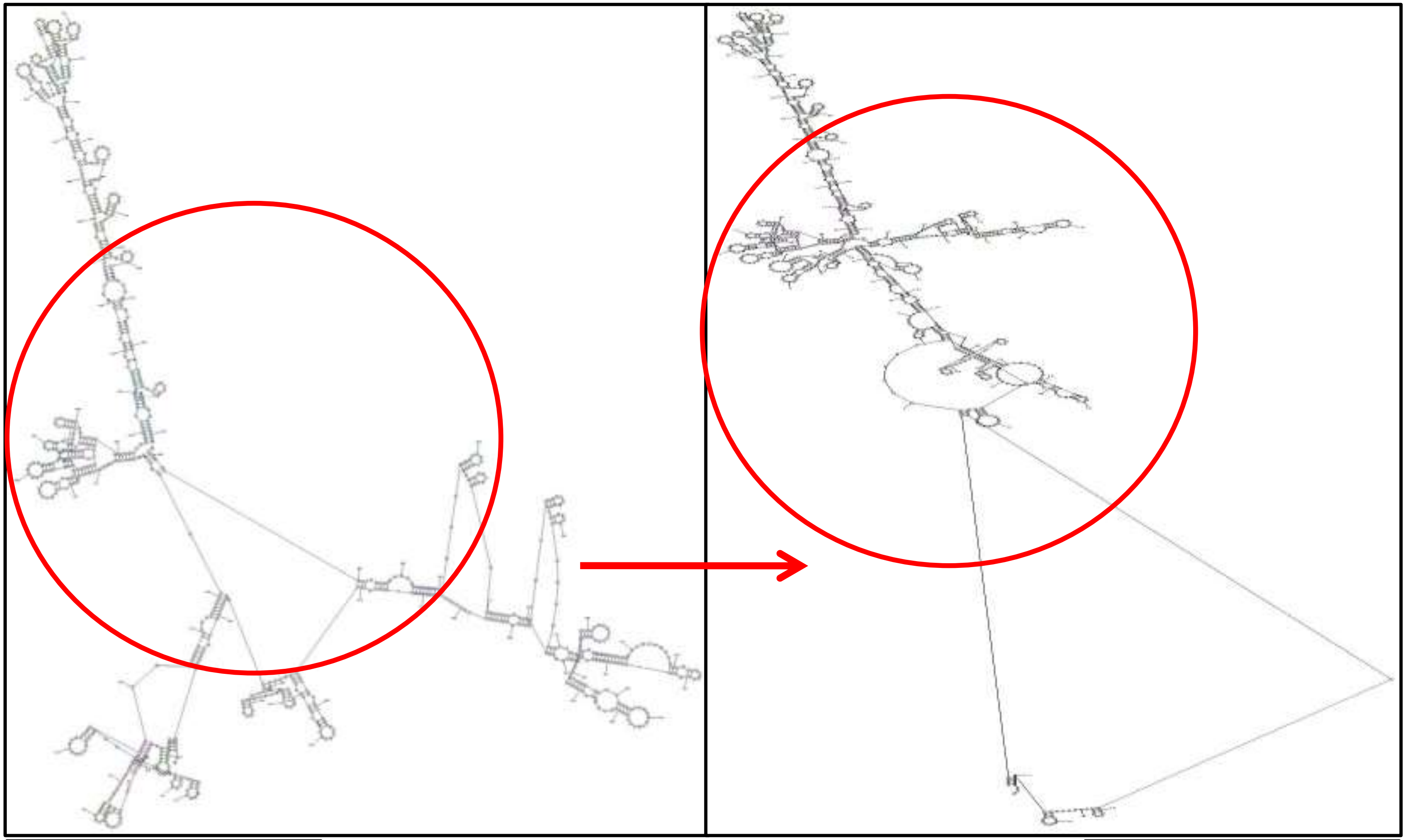

Energy= -187.8 [WT]

Energy= -190.0 [MT]

rs3134942 A>C_*NOTCH4*

Supplementary Figure 3 [SF3]: Pictorial representation of change in secondary structure of mRNA of *NOTCH4* gene due to rs3134942wild-type [WT] and mutant [MT] variants.

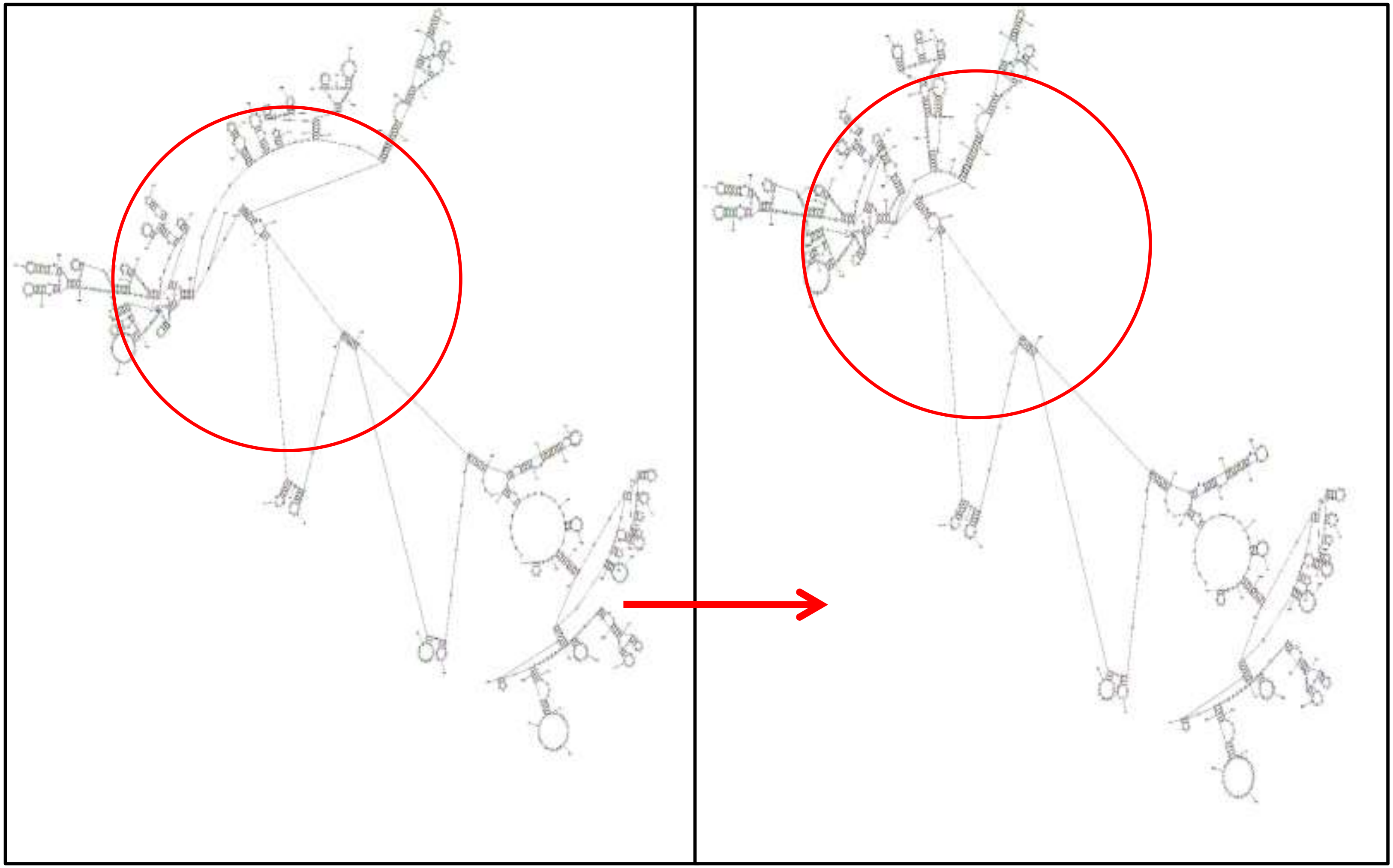

Energy= -145.6 [WT]

rs291700 C>T_*CDK5RAP1*

Energy= -148.4 [MT]

Supplementary Figure 4 [SF4]: Pictorial representation of change in secondary structure of mRNA of *CDK5RAP1* gene due rs291700 wild-type [WT] and mutant [MT] variants.
