# Supplementary material for: A comprehensive meta-analysis and prioritization study to identify vitiligo associated coding and non-coding SNV candidates using web-based bioinformatics tools
Source: Sci Rep. 2022 Aug 25;12:14543. doi: 10.1038/s41598-022-18766-9 (PMC9411560; doi:10.1038/s41598-022-18766-9)
Supplement: Supplementary file 2 — Supplementary Information 2. [file 41598_2022_18766_MOESM2_ESM.pdf]

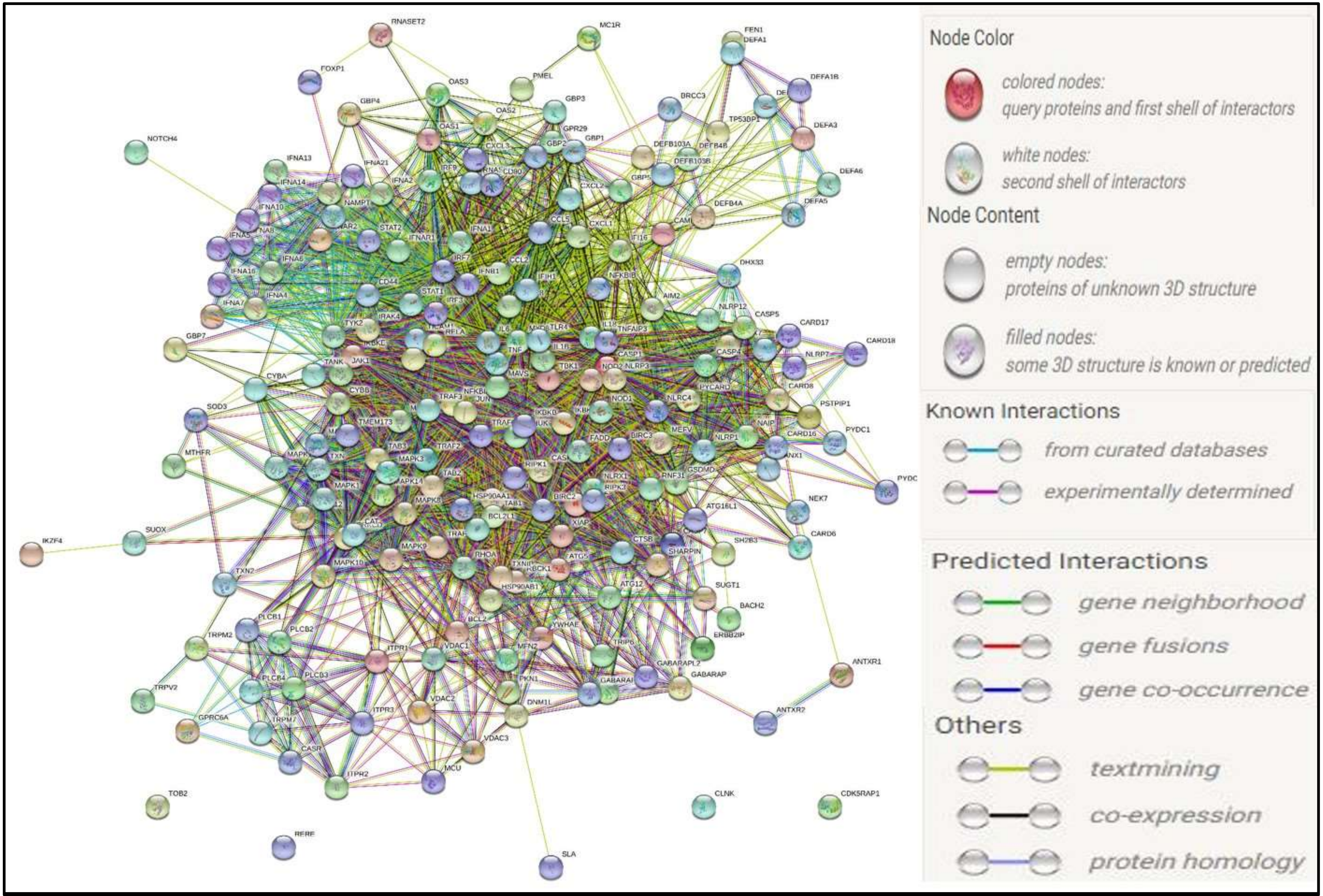

Supplementary Figure 2: *Pictorial representation of interactions found among our prioritized gene and well-known validated vitiligo gene dataset using STRING v.11.0*
